# Winter Exercise Reduces Allergic Airway Inflammation: A Randomized Controlled Study

**DOI:** 10.3390/ijerph16112040

**Published:** 2019-06-08

**Authors:** Johanna Prossegger, Daniela Huber, Carina Grafetstätter, Christina Pichler, Herbert Braunschmid, Renate Weisböck-Erdheim, Arnulf Hartl

**Affiliations:** 1 Institute of Ecomedicine, Paracelsus Medical University Salzburg, 5020 Salzburg, Austria; johanna.prossegger@pmu.ac.at (J.P.); daniela.huber@fh-salzburg.ac.at (D.H.); c.grafetstaetter@pmu.ac.at (C.G.); christina.pichler@pmu.ac.at (C.P.); herbert.braunschmid@stud.sbg.ac.at (H.B.); renate.erdheim@pmu.ac.at (R.W.-E.); 2 Department of Physiotherapy, Salzburg University of Applied Sciences, 5412 Puch/Urstein, Austria

**Keywords:** allergy, allergic rhinitis, asthma, exercise, exhaled nitric oxide

## Abstract

*Background:* Physical exercise is often recommended as additional treatment for people suffering from allergic rhinitis and/or asthma, but less is known about the specific effects of recreational winter outdoor exercise on allergic airway inflammation. *Methods:* We performed a longitudinal, randomized controlled intervention study to investigate the effects of recreational winter exercise on allergic airway inflammation, quality of life, spirometry and cardiorespiratory fitness in adults suffering from allergic rhinitis and/or asthma. The exercise group participated in a ten-day winter sports program. The control group did not receive any intervention. *Results:* A significant improvement of fractional oral exhaled nitric oxide (FeNO; *p* = 0.008, day 10) and a significant decrease in FeNO after a single 4 h hiking tour (*p* < 0.001, time effect) were observed for the exercise group. The nasal eosinophilic cell count revealed a short-term reduction (*p* = 0.021, treatment effect) in the exercise group and for the visual analogue scale sustainable improvements in allergic symptoms (*p* < 0.001, day 60) were found. No adverse effects of outdoor winter exercise were observed. *Conclusion:* Recreational winter exercise at moderately cold temperatures reduces allergic airway inflammation measured as FeNO, nasal eosinophilic cell count and induces sustainable improvements in allergic symptoms.

## 1. Introduction

The prevalence of allergies and asthma has increased during the last decades. The World Health Organization (WHO) estimates that 400 million people in the world suffer from allergic rhinitis, and 300 million from asthma. Respiratory allergies affect around 20%–30% of the European population [1,2]. Traditionally, allergic rhinitis and allergic asthma have been treated as two distinct diseases, but many epidemiologic studies have highlighted the link between the two diseases [3,4,5]. Following the concept of the united airway disease, an integrative perspective of allergic rhinitis and asthma is mandatory to achieve optimal treatment effects [6,7,8]. Allergies have become a serious economic burden as allergic diseases not only affect the quality of life negatively, but also lead to reduced work productivity [9,10]. Asthma and allergies are one of the most common chronic diseases in children and the leading causes of school absences, chronic medication usage and hospitalizations [11]. Facing this development, allergic diseases should be recognized as a serious public health problem and efforts towards optimal treatments should be made.

The conservative treatment of allergic rhinitis and/or asthma includes several pharmacological derivates like antihistamines, bronchodilators or glucocorticoids [12,13]. Apart from these pharmacological approaches, traveling and staying at high altitudes have a long tradition and have been described to reduce symptoms [14,15]. Also, regular physical activity and sports have been shown to improve asthma symptoms, quality of life and exercise capacity [16]. Although physical exercise is a well-known trigger for asthma symptoms, physical inactivity increases asthma-related difficulties. Exercise avoidance leads to a reduction of exercise capacity, thus lowering the threshold for triggering symptoms [17,18]. Patients with stable asthma should therefore be encouraged to participate in regular physical exercise. 

Most studies regarding physical training for asthmatics include swimming, treadmill or aerobic exercise training. Although training outdoors in natural environments results in significantly greater feelings of enjoyment and satisfaction, and people are more likely to continue exercising [19,20], scientific evidence towards outdoor exercise training for people with allergies and asthma, especially for winter exercise, seems to be rare [21]. Exercising outdoors in winter may improve vitamin D levels, as sunlight is required for its production. Vitamin D deficiency is still common among adults, especially during the winter months [22,23], and vitamin D seems to play a crucial role in the prevention and treatment of allergic rhinitis and/or asthma [24,25]. Considering that 31% of the world’s population are not meeting the minimum recommendations for physical activity [26] and the fact that people tend to spend more time indoors during the winter months [27], not only patients suffering from allergic rhinitis and/or asthma, but also healthy people should be encouraged to maintain an active lifestyle during the winter.

With the decrease of temperature in the winter, the heating period starts and the fine dust pollution in urban areas increases significantly. As a consequence of atmospheric inversion, smoke, diesel exhaust and fine dust particles are trapped in lower atmospheric strata, wrapping cities in a grey dust coat [28]. The alpine regions are like an island in a sea of grey fine-dust pollution [29], offering not only perfect conditions for diverse winter sports, including skiing, ski mountaineering, cross-country skiing and winter hiking, but also clean and fresh air.

Sports and physical activity are considered to be cost-effective methods to improve public health [30], even for people suffering from allergic rhinitis and/or asthma [31]. However, the current Global Initiative for Asthma guidelines still do not include recommendations for physical activity [32]. Evidence-based guidelines are missing for recreational winter sports for patients with allergic rhinitis and/or asthma. Most research is focused on elite athletes and therefore patients have to rely on personal recommendations from their physicians [33]. This can be difficult sometimes, as guidelines are missing. In this randomized controlled clinical study, we investigated the specific effects of recreational outdoor winter exercise on allergic airway inflammation, quality of life, spirometry, cardiorespiratory fitness and allergic symptoms on people suffering from allergic rhinitis and/or asthma.

## 2. Materials and Methods

### 2.1. Study Design and Settings

The presented data is a subsection from the WESPAA study (Winter Exercise and Speleotherapy for Allergy and Asthma; ISRCTN88277657), a longitudinal, randomized, controlled intervention study, which investigated the effects of winter exercise in combination with speleotherapy and winter exercise on patients suffering from allergic rhinitis and/or allergic asthma. The allocation ratio for the control group and the two intervention groups was set at equal sample size. The study protocol was approved by the Ethics Committee of Salzburg (415-E/1553/2-2012) and was conducted in the holiday region of Hohe Tauern National Park (Salzburg, Austria) between March and December 2013. To ensure a clear and simple data presentation, the study results were divided into two sections: specific effects of winter exercise and combined effects of winter exercise and speleotherapy. This work is focused on the specific effects of winter exercise solely and presents only data from the exercise and control group. Further publications will focus on the combined effects of winter exercise and speleotherapy. 

### 2.2. Participants

Adults between 18 and 55 years with a house dust mite sensitization and allergic asthma and/or rhinitis were recruited from Austria and Germany through ads and a website between November 2012 and February 2013. Inclusion criteria were as follows: aged 18–55 years, house dust mite sensitization (radioallergosorbent test (RAST) >1; positive PRICK test or total immunoglobulin E (IgE) > 0.7 kU/l), controlled allergic rhinitis and/or allergic asthma and the physical ability—including moderate skiing skills—to meet the demands of the exercise program. Exclusion criteria were as follows: uncontrolled asthma (asthma control test <20) [34], malignant neoplastic disorders, exercise induced bronchoconstriction, cardiovascular diseases, orthopedic diseases, lung function disorder, acute infection or fever, uncontrolled metabolic diseases and pregnancy.

### 2.3. Intervention

The participants of the exercise group spent a ten-day holiday in December 2013 in Hollersbach, Austria (47°16’35.4” N 12°25’09.8” E) located 806 m above sea level. All participants were hosted in certified “allergy friendly hotels” and received the same meals. To refresh basic knowledge about asthma self-management and safety aspects, the participants of the intervention groups participated in a one-hour asthma class with a physiotherapist. The exercise program was composed of four 3–5 h guided GPS-monitored hiking/snow-shoe tours with an average altitude difference of 411 m and 11 km in distance per day and four all day skiing sessions with an average of 42 km in ski slopes in three different ski regions (2.000–2.500 m). The control group did not receive any intervention. Assessments of the control group were performed together with the speleotherapy group between March and May 2013.

### 2.4. Data Collection and Outcomes

**Figure 1:**
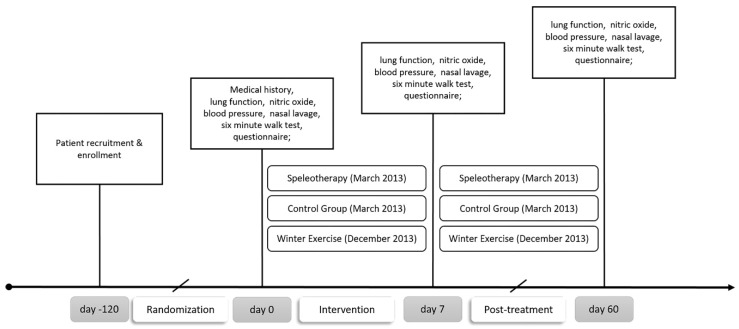
Study schedule. This work is focused on winter exercise solely. No data from the speleotherapy group are shown.

All medical examinations were performed in a mobile lab setup at the hospital in Mittersill and at the Paracelsus Medical University in Salzburg. Data were anonymized by a four-digit ID. Primary outcomes were fractional exhaled nitric oxide (FeNO) and the German version of the RhinAsthma Quality of Life Scale [35]. Secondary outcomes were spirometry, differential blood count, eosinophilic cell count from nasal lavage, six-minute walk test (6MWT), mucociliary clearance time and inverse visual analogue scale concerning health status and allergy symptoms (a higher value indicates a better clinical result). Assessments were performed at baseline (day 0; T0), after the intervention (day 10; T1) and after two months (day 60, T2). To assess short-term effects of a single winter hike, three additional FeNO measurements were performed at day 9. No specific lifestyle recommendations were given for the non-treatment period in any group. The study schedule is presented in Figure 1. At each time point, 12 mL of forearm venous blood were collected in tubes (BD Vacutainer® system (Becton Dickinson AG, Vienna, Austria) according to the manufacturer’s guidelines. Differential blood count was performed by the University Institute for Medical and Chemical Laboratory Diagnostics of the Paracelsus Medical University Salzburg (Austria).

#### 2.4.1. Fractional Exhaled Nitric Oxide

Fractional exhaled nitric oxide (FeNO) was measured by NioxMino® (Aerocrine AB, Solna, Sweden) according to the ATS/ERS guidelines [36]. Due to technical problems with the NioxMino®, data from 9 participants from the exercise group were lost at baseline. Statistical analysis was performed with the remaining 9 participants, which completed the full time series. To assess the effects of a single winter hike, additional FeNO measurements were performed in the exercise group before a winter hike on day 9, after a 4 h hiking tour, after 1 hour of rest and 24 h later on day 10 (T1).

#### 2.4.2. Spirometry and Six-Minute Walk Test

Spirometry was performed using the EasyOne spirometer (ndd Medical Technologies, Zurich, Switzerland) with a forced ex-in maneuver, according to manufacturer’s and ATS/ERS guidelines [37]. Respiratory parameters analyzed were as follows: forced expiratory volume (FVC) (%), forced expiratory volume in 1 second (FEV_1_) (%), forced expiratory volume in 1 second of forced vital capacity (FEV_1_/FVC) (%), peak expiratory flow (PEF) (%) and mid-expiratory flow at 25%–75% of the FVC (MEF_25%_–_75%_) (%).

The six-minute walk test (6MWT) was performed according to manufacturer’s and ATS guidelines [38] with a SpiroPalm 6MWT device (COSMED, Rome, Italy). The following parameters were analyzed: distance walked in 6 minutes (%), BORG scale for fatigue and dyspnea, peak minute ventilation (L/min) and peak ventilation frequency (1/min). Reference values for the predicted distance are described elsewhere [39].

#### 2.4.3. Nasal Lavage and Saccharin Test

Nasal lavage (NAL) was performed with 5 mL warm PBS (22–25 °C). A sample of at least 3 mL nasal lavage secretion was collected and filled into a precooled, sterile container. Before centrifugation (3800 rpm, 15 min, room temperature), samples were vigorously shaken. The pellet was suspended in PBS (phosphate bufferd saline). For each participant two cell smears were obtained by low-speed centrifugation (400 rpm, 6 min, room temperature) with a Cytospin 3 (Thermo Fischer, Waltham, Massachusetts, USA). The smears were stained with Giemsa stain (Sigma Giemsa Stain, Sigma-Aldrich Handels GmbH, Vienna, Austria) and mounted with Entellan® (Sigma-Aldrich Handels GmbH, Vienna, Austria). Cells were counted under light microscopy at 100× magnification. Cell counts were performed on both smears, with a total count of at least 200 cells per smear.

Mucociliary clearance time was measured via the saccharin test described by Andersen et.al. [40]. A saccharin particle was placed in the inferior turbinate of one nasal cavity at least 7 mm behind the anterior end of the turbinate. The participants were instructed to sit down and not to sniff, sneeze, cough, eat or drink.

### 2.5. Randomization and Sample Size

Randomization was performed using open-source add-in (Daniel’s XL Toolbox, 2012, Daniel Kraus, Würzburg, Germany) for the Microsoft Excel® spreadsheet software, using the Kullback–Leibler divergence method [41]. Recruitment of eligible participants, randomization and assignment to treatments were performed by the same researcher. No sample size calculation was performed.

### 2.6. Statistical Analysis

All statistical analyses were performed using the R-GNU software environment (General Public License, R Foundation for Statistical Computing, version 3.4.4, Vienna, Austria). Statistical significance was set at the 0.05 probability level for all tests. All variables were expressed as mean ± standard deviation unless otherwise indicated. All results were corrected for multiple comparisons (exercise vs. control and speleotherapy vs. control), according to the Holm–Bonferroni method. Longitudinal data analysis was performed using the nparLD package [42], which offers a fully nonparametric analysis of variance-type testing. In the F1-LD-F1 model, treatment was included (exercise vs. control) as whole-plot factor and time as sub-plot factor (T0, T1 and T2). Post-hoc tests were applied in case of a significant main effect for time with another F1-LD-F1 model. For the longitudinal analysis of the short-term effects of FeNO a LD-F1 model with time as the sub-plot factor was applied. As a measure of effect, relative treatment effects (RTEs) were used. 

The RTE is a measure of effect and can be interpreted as following: an RTE of 0.2 for a subgroup means that the probability of a randomly chosen person from the whole subset having a lower score than a randomly chosen person from the subset is estimated to be 20%. An RTE of 0.5 means no effect. An RTE > 0.5 means a tendency for subjects in a subgroup to score at least as high as a randomly chosen subject from the whole sample.

## 3. Results

### 3.1. Study Participants and Baseline Characteristics

**Figure 2:**
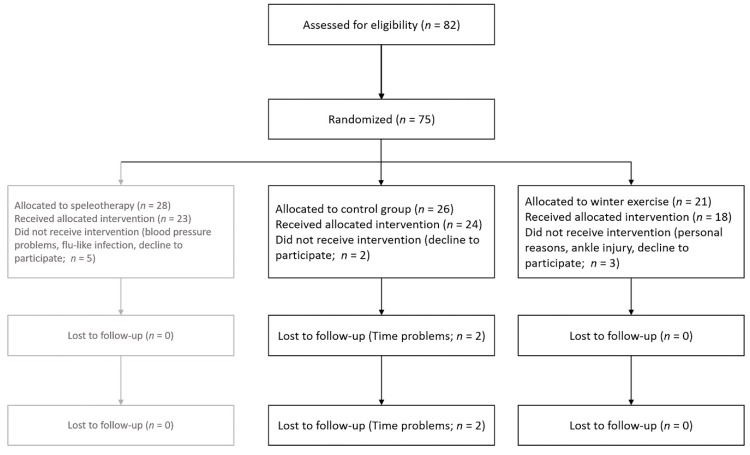
Study flowchart of included and excluded patients.

Out of 75 eligible people, 26 people were enrolled for the control group in March 2013 and 21 people were invited for the winter exercise program in December 2013. Two participants from the control group and one from the exercise group declined to participate after enrollment. Two participants from the control group were lost during follow-up because of limited time resources. One participant from the exercise group quit the study due to personal reasons and one person quit due to a strain in the ankle. For the per-protocol analysis 18 participants of the exercise group and 22 participants of the control group (Figure 2) were included.

Baseline characteristics showed no relevant differences between the study groups (Table 1), except for age. Descriptive statistics over all time points are summarized in Tables S1 and S2 in the Supplementary Materials. The exercise group was significantly older than the control group (Mann–Whitney U Test, *p* = 0.021, W = 112.5). The winter exercise program was well tolerated by all participants. No harms or unintended effects like further injuries, exercise-induced bronchoconstriction or severe asthma symptoms were observed. Ambient air temperature for the exercise program ranged between −5.6 °C to 8.7 °C (air temperature 2 m above ground; Zentralanstalt für Meteorologie und Geodynamik, Austria).

### 3.2. Fractional Exhaled Nitric Oxide and Spirometry

The analysis of variance type test for oral FeNO revealed no significant main effect for treatment or the interaction. A trend within the main effect time (*p* = 0.055) was observed and post-hoc tests revealed a significant interaction effect (*p* = 0.008) on day 10, indicating a short-term reduction of FeNO in the exercise group. No significant changes were found for nasal FeNO, although the RTEs indicate a decrease in the exercise group (see Table 2). 

Spirometry, including FVC (%), FEV_1_ (%), FEV_1_/FVC (%), PEF (%) and MEF_25%_–_75%_ (%), revealed no significant changes. For FEV_1_/FVC (%) and PEF, a trend in the main effect for time was observed, but post-hoc tests did not reveal any further significances (Table 2).

### 3.3. Short-Term Effects of Winter Hiking on FeNO

**Figure 3:**
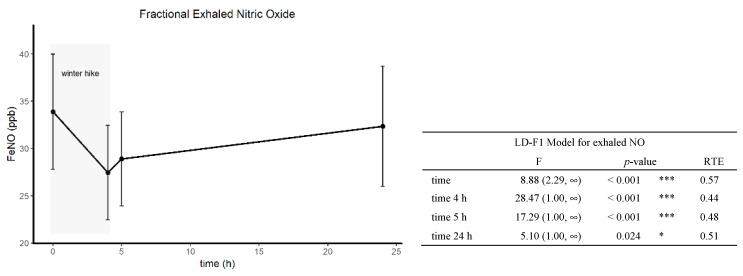
Short-term effects of a single winter hike (4 h) on oral FeNO at day 9. F1-LD-F1 model with time and treatment (treat, exercise or control) and the interaction of treatment and time (treat x time); T0, T1 and T2 time points T0 = day 0, T1 = day 10, T2 = day 60; ** < 0.01; * <0.005; <0.1; n.s. not significant.

The analysis of the short-term effects of winter hiking on FeNO with a non-parametric LD-F1 model revealed a significant time effect (*p* < 0.001), indicating a decrease in FeNO. Post-hoc tests showed significant effects at all time points (after the hike *p* < 0.001; after 1 h rest *p* < 0.001; after 24 h *p* = 0.024, Figure 3).

### 3.4. RhinAsthma Quality of Life Questionnaire (German adapted version)

The analysis of variance type test for the total score of the German version of the RhinAsthma questionnaire revealed a significant main effect for time, but post-hoc tests did not show any interaction effects at the single time points. The analysis of the subscales revealed no significant changes for “limitations in daily life”, “respiratory problems”, “treatment and medication problems” and “impairment in sensory perception”. For the “rhinoconjunctivitis score” a trend within the main effect for the interaction (*p* = 0.058) was found and RTEs indicate a short-term improvement in the exercise group (Table 3).

### 3.5. Nasal Eosinophilic Count and Mucociliary Clearance Time

The nasal eosinophil count revealed a significant main effect for the interaction (*p* = 0.021), but no treatment or time effects were observed (Table 2). No significant changes were found for the mucociliary clearance time, although the RTEs indicate a short-term reduction in the exercise group (Table 2). 

### 3.6. Differential Blood Count

No significant changes were found for the differential blood count including red blood cell count, neutrophils and eosinophils. The white blood cell count presented a significant baseline difference (Mann–Whitney U Test, *p* < 0.001, W = 81.5). The exercise group was characterized by a higher white blood cell count (7.41 ± 1.30 10³/µL) in comparison to the control group (5.48 ± 1.51 10³/µL). The analysis of variance type test for the white blood cell count revealed a significant main effect of treatment (*p* = 0.007) and interaction (*p* = 0.004, see Table 2).

### 3.7. Six-Minute Walk Test

The analysis of the predicted distance in the 6MWT revealed a significant main effect of time (*p* = 0.009). Post-hoc tests did not show any significances. For the peak respiratory frequency, a significant main interaction effect (*p* = 0.015) was found. The analysis of the peak minute ventilation showed no significant treatment or interaction effect. A significant time effect (*p* = 0.012) was observed, but post-hoc tests did not reveal any significant interaction effects. No significant changes were observed for the BORG scale (Table 4).

### 3.8. Visual Analogue Scale

The analysis of variance type test for the visual analogue scale for allergic symptoms revealed a significant main effect of time (*p* = 0.002) and of interaction (*p* < 0.001). Post-hoc tests showed a significant interaction effect on day 10 (*p* = 0.005) and on day 60 (*p* < 0.001), indicating sustainable improvements in allergic symptoms. The visual analogue scale for general health presents a significant baseline difference (Mann–Whitney U Test, *p* = 0.01, W = 289). The exercise group (71.66 ± 14.10%) is characterized by a lower score in comparison to the control group (80.77 ±. 17.82%, see Table 3). Only a significant main effect of the interaction (*p* = 0.009) was found.

F1-LD-F1 model with time and treatment (treat, exercise or control) and the interaction of treatment and time (treat × time); T0, T1 and T2 time points T0 = day 0, T1 = day 10, T2 = day 60; *** <0.001; ** < 0.01; * <0.005; <0.1; n.s. not significant. 

## 4. Discussion

Allergic respiratory diseases have become a serious public health problem. Although physical exercise is a well-known and often recommended additional treatment, the effectiveness of physical training on allergic airway inflammation is still under discussion [43]. The aim of this randomized controlled clinical study was to examine whether moderate winter exercise improves allergic airway inflammation and quality of life in patients suffering from allergic rhinitis and/or asthma. Additionally, the effects on spirometry, differential blood count, eosinophilic cell count from nasal lavage, six-minute walk test and allergic symptoms were examined. A significant reduction of FeNO and nasal eosinophilic cell count as well as sustainable improvements in allergic symptoms were found in the exercise group.

Within the general population, physical activity is associated with an improved health status [44]. This also applies to people who suffer from respiratory allergies. Physically active patients have fewer exacerbations, lower FeNO levels and less symptoms compared to those with a sedentary lifestyle [45]. However, people suffering from asthma are considered to be less active than their healthy peers [46,47]. The fear of triggering symptoms, but also the amount of ambient air pollution can be a limiting factor for outdoor exercise [48]. The holiday region of Hohe Tauern National Park in Salzburg, Austria, is characterized by low levels of air pollution [29,49] and offers an ideal environment for winter outdoor exercise for this vulnerable patient group.

Fractional exhaled NO is a reliable surrogate parameter measuring allergic airway inflammation [50]. Acute moderately intensive exercise is associated with a decrease in FeNO in physically inactive adults with asthma [51]. Additionally murein models provide strong evidence that physical training reverses airway remodeling and improves allergic airway inflammation [52]. Within the winter exercise group, oral exhaled FeNO decreased. Although, nasal exhaled FeNO did not reach statistical significance, the RTEs also indicated a decrease. Our results clearly show that recreational outdoor winter exercise at moderately cold temperatures has a beneficial effect on the allergic airway inflammation in patients suffering from allergic rhinitis and/or asthma. Although data from nine participants of the exercise group were lost at baseline, the statistical analysis revealed a significant decrease of FeNO for the remaining participants. Furthermore, a single 4 h winter hiking tour at day 9 induced a significant short-term reduction of FeNO. The timing for the evaluation of the immediate effects at day 9 is admittedly suboptimal, but technical problems made it impossible to evaluate this effect on day 1. However, as FeNO decreased significantly in both situations, it can be assumed that recreational outdoor winter exercise at moderately cold temperatures (−5.6 °C to 8.7 °C) has a beneficial effect on allergic airway inflammation. 

Regular physical exercise is considered to improve the quality of life in patients suffering from allergic rhinitis and/or asthma [53]. Although, the results from the RhinAsthma quality of life questionnaire do not show a significant improvement in the total score. A statistical trend and the relative treatment effects of the exercise group (see Table 3) point towards a short-term improvement of the subscale rhinoconjunctivitis score. The inverse visual analogue scale even reveals a sustainable improvement of allergic symptoms. No significant changes were found for mucociliary clearance time, although the relative treatment effects indicate a short-term improvement in the exercise group. No effects were found for spirometry, differential blood count and the 6MWT distance. Although a significant effect was found for the peak respiratory frequency, the RTEs show a rather small treatment effect. The 6MWT distance is a simple surrogate parameter for cardio-respiratory fitness [54]. Both groups presented high 6MWT distances (exercise group 104.35 ± 8.17%; control group 103.36 ± 10.49%) at baseline. As one inclusion criterion was the physical constitution for recreational winter exercise, no significant improvements in the 6MWT distance were expected for a short intervention time of ten days.

### Strengths and Limitations

Due to a high supervisor–participant ratio during the exercise program, it was not possible to carry out the control and intervention groups simultaneously. To cope with the small number of participants, a crossover design could have been used instead of a parallel study design. All statistical analyses were performed using rank-based nonparametric methods with ANOVA-type statistics to accurately control the type I error rate within this small sample size. The ambient air temperatures in December and March are quite comparable in the study region, therefore a bias from different environmental conditions can be excluded. The exercise group was significantly older than the control group, but age is not known to be a major confounder for allergic respiratory disease. Even though the control group did not receive any treatment, two distinct study settings were created. The participants of the control group remained in their personal and professional daily routine with higher levels of ambient air pollution and stress and less exercise in comparison to the exercise group. This study design allows for comparison of a ten-day holiday with recreational winter exercise with real life conditions and the daily routine of people suffering from allergic rhinitis and/or asthma. As the sample represents only a small part of the disease severity spectrum, further studies are needed for detailed recommendations on outdoor winter exercise for people with allergic rhinitis and/or asthma.

## 5. Conclusions

To our knowledge this is the first clinical study focusing on the effects of recreational winter exercise on patients suffering from allergic rhinitis and/or asthma. A ten-day winter sports program reduced the allergic airway inflammation in patients suffering from allergic rhinitis and/or asthma. Furthermore, the number of eosinophilic cells from nasal lavage decreased and allergic symptoms improved, thus indicating an overall improvement of the inflammatory milieu in the airways. None of the participants reported undesirable effects during measurements or the winter exercise program. In contrast, all patients showed high compliance and good physical tolerance. Recreational outdoor winter exercise may be therefore recommended for patients with good disease control. 

## Figures and Tables

**Table 1:** Baseline characteristics of the study population.

	Exercise Group (*n* = 18)		Control Group (*n* = 22)	
	Mean ± SD	Median ± IQR	Mean ± SD	Median ± IQR
Gender	male *n* = 5	female *n* = 13	male *n* = 7	female *n* = 15
Residence *	rural *n* = 5	urban *n* = 13	rural *n* = 9	urban *n* = 13
RAST/PRICK	2 or + *n* = 8	>3 or > ++ *n* = 10	2 or + n = 10	>3 or > ++ *n* = 12
Age (years)	40.6 ± 12.1	45.0 ± 22.3	31.6 ± 10.7	25.5 ± 18.3
Height (m)	172.6 ± 6.7	172.0 ± 7.5	172.7 ± 8.1	171.5 ± 10.8
Weight (kg)	69.8 ± 9.8	69.0 ± 10.0	66.9 ± 9.3	67.5 ± 13.3
Pulse (bpm)	71.0 ± 12.6	70.4 ± 23.0	68.7 ± 8.8	67.0 ± 10.8
BP-Systole (mmHg)	125.3 ± 14.5	123.5 ± 14.5	118.5 ± 12.4	117.0 ± 20.0
BP-Diastole (mmHg)	76.4 ± 10.0	78.0 ± 15.0	73.0 ± 10.2	70.0 ± 17.8
ACT (score)	21.4 ± 3.6	22.5 ± 4.5	22.9 ± 3.2	24.0 ± 2.8
FeNO (ppb)	40.8 ± 23.0	38.0 ± 39.0	38.9 ± 22.3	36.0 ± 22.5
FEV1 (%)	96.3 ± 21.0	98.5 ± 21.3	103.7 ± 13.2	99.5 ± 18.5
FVC (%)	108.2 ± 17.7	108.0 ± 12.8	110.3 ± 16.1	109.0 ± 24.5
FEV1/FVC (%)	93.4 ± 11.8	95.0 ± 17.0	97.7 ± 8.2	99.5 ± 9.8
6MWT Distance (%)	104.4 ± 8.2	106.0 ± 11.0	103.4 ± 10.5	102.6 ± 15.8

* number of participants living in an urban/rural environment; urban >10,000 citizens. ++ is threating system of the PRICK test; IQR, interquartile range; RAST, radioallergosorbent test; PRICK, allergy-prick test; BP: blood pressure; ACT: asthma control test; FEV_1_: forced expiratory volume in 1 second; FVC: forced expiratory volume; FEV_1_/FVC: forced expiratory volume in 1 second of forced vital capacity; 6MWT: six-minute walk test.

**Table 2:** Results from the F1-LD-F1 model for FeNO, nasal eosinophilic count, mucociliary clearance time, spirometry and differential blood count.

Parameter	F1-LD-F1 Model				Relative Treatment Effects							
		F	*p*-value				Time		Interaction Effects			
Oral FeNO (ppb)	Treat	1.15 (1.00, ∞)	1.000	n.s	Exercise	0.42	T0	0.54	Exercise × T0	0.55	Control × T0	0.52
	Time	4.39 (1.63, ∞)	0.055		Control	0.53	T1	0.39	Exercise × T1	0.29	Control × T1	0.49
	Treat × Time	3.17 (1.63, ∞)	0.261	n.s			T2	0.50	Exercise × T2	0.41	Control × T2	0.58
	Treat × T1	10.23 (1.0, ∞)	0.008	**								
Nasal FeNO (ppb)	Treat	0.37 (1.00, ∞)	1.000	n.s	Exercise	0.46	T0	0.52	Exercise × T0	0.55	Control × T0	0.48
	Time	0.65 (1.83, ∞)	0.509	n.s	Control	0.52	T1	0.44	Exercise × T1	0.40	Control × T1	0.47
	Treat × Time	0.99 (1.83, ∞)	0.733	n.s			T2	0.51	Exercise × T2	0.44	Control × T2	0.59
Nasal Eosinophil Count (%)	Treat	0.08 (1.00, ∞)	1.000	n.s	Exercise	0.51	T0	0.50	Exercise × T0	0.57	Control × T0	0.42
	Time	2.40 (1.98, ∞)	0.183	n.s	Control	0.49	T1	0.46	Exercise × T1	0.45	Control × T1	0.47
	Treat × Time	3.90 (1.98, ∞)	0.021	*			T2	0.55	Exercise × T2	0.52	Control × T2	0.58
Mucociliary Clearance Time (min)	Treat	0.50 (1.00, ∞)	0.754	n.s	Exercise	0.47	T0	0.52	Exercise × T0	0.50	Control × T0	0.55
	Time	3.64 (1.8,1 ∞)	0.061	n.s	Control	0.52	T1	0.44	Exercise × T1	0.38	Control × T1	0.50
	Treat × Time	1.88 (1.81, ∞)	0.470	n.s			T2	0.53	Exercise × T2	0.53	Control × T2	0.52
FVC (%)	Treat	0.06 (1.00, ∞)	1.000	n.s	Exercise	0.49	T0	0.50	Exercise × T0	0.47	Control × T0	0.52
	Time	0,65 (1.81, ∞)	1.000	n.s	Control	0.51	T1	0.49	Exercise × T1	0.48	Control × T1	0.51
	Treat × Time	1,92 (1.81, ∞)	0.303	n.s			T2	0.51	Exercise × T2	0.51	Control × T2	0.50
FEV_1_	Treat	0.89 (1.00, ∞)	0.689	n.s	Exercise	0.45	T0	0.49	Exercise × T0	0.44	Control × T0	0.54
	Time	1.58 (1.81, ∞)	0.416	n.s	Control	0.54	T1	0.51	Exercise × T1	0.47	Control × T1	0.55
	Treat × Time	0.49 (1.81, ∞)	1.000	n.s			T2	0.48	Exercise × T2	0.45	Control × T2	0.52
FEV_1_/FVC (%)	Treat	1.71 (1.00, ∞)	0.585	n.s	Exercise	0.43	T0	0.49	Exercise × T0	0.43	Control × T0	0.55
	Time	4.10 (1.85, ∞)	0.076		Control	0.55	T1	0.52	Exercise × T1	0.47	Control × T1	0.56
	Treat × Time	2.61 (1.85, ∞)	0.313	n.s			T2	0.47	Exercise × T2	0.40	Control × T2	0.55
PEF (%)	Treat	0.05 (1.00, ∞)	1.000	n.s	Exercise	0.49	T0	0.46	Exercise × T0	0.48	Control × T0	0.45
	Time	2.70 (1.98, ∞)	0.068		Control	0.51	T1	0.52	Exercise × T1	0.48	Control × T1	0.56
	Treat × Time	2.59 (1.88, ∞)	0.302	n.s			T2	0.51	Exercise × T2	0.51	Control × T2	0.52
MEF _25%-75%_	Treat	1.94 (1.00, ∞)	0.328	n.s	Exercise	0.43	T0	0.49	Exercise × T0	0.41	Control × T0	0.56
	Time	2.73 (1.76, ∞)	0.145	n.s	Control	0.56	T1	0.52	Exercise × T1	0.47	Control × T1	0.56
	Treat × Time	2.00 (1.76, ∞)	0.284	n.s			T2	0.48	Exercise × T2	0.41	Control × T2	0.55
WBC (10³/µL)	Treat	9.16 (1.00, ∞)	0.005	**	Exercise	0.62	T0	0.52	Exercise × T0	0.67	Control × T0	0.36
	Time	0.27 (1.98, ∞)	0.758	n.s	Control	0.40	T1	0.49	Exercise × T1	0.52	Control × T1	0.47
	Treat × Time	6.31 (1.98, ∞)	0.002	**			T2	0.52	Exercise × T2	0.66	Control × T2	0.38
RBC (10^6^/µL)	Treat	0.24 (1.00, ∞)	0.622	n.s	Exercise	0.52	T0	0.53	Exercise × T0	0.54	Control × T0	0.53
	Time	1.35 (1.93, ∞)	0.520	n.s	Control	0.48	T1	0.49	Exercise × T1	0.52	Control × T1	0.45
	Treat × Time	0.40 (1.93, ∞)	1.000	n.s			T2	0.49	Exercise × T2	0.51	Control × T2	0.46
Blood Neutrophil count (%)	Treat	0.65 (1.00, ∞)	0.419	n.s	Exercise	0.54	T0	0.51	Exercise × T0	0.59	Control × T0	0.44
	Time	0.16 (1.57, ∞)	0.798	n.s	Control	0.47	T1	0.50	Exercise × T1	0.52	Control × T1	0.48
	Treat × Time	2.25 (1.57, ∞)	0.235	n.s			T2	0.50	Exercise × T2	0.51	Control × T2	0.49
Blood Eosinophil count (%)	Treat	0.01 (1.00, ∞)	0.907	n.s	Exercise	0.49	T0	0.50	Exercise × T0	0.51	Control × T0	0.49
	Time	0.18 (1.62, ∞)	0.787	n.s	Control	0.50	T1	0.49	Exercise × T1	0.50	Control × T1	0.48
	Treat × Time	1.21 (1.62, ∞)	0.292	n.s			T2	0.51	Exercise × T2	0.48	Control × T2	0.54

F1-LD-F1 model with time and treatment (treat, exercise or control) and the interaction of treatment and time (treat × time); T0, T1 and T2 time points T0 = day 0, T1 = day 10, T2 = day 60; ** <0.01; * <0.005; <0.1; n.s. not significant. Abbreviations: PEF, peak expiratory flow; MEF _25%–75%_, mid-expiratory flow at 25%–75% of the FVC; WBC, white blood cell count; RBC, red blood cell count.

**Table 3:** Results from the F1-LD-F1 model for questionnaires.

Parameter	F1-LD-F1 Model				Relative Treatment Effects							
		F	*p*-value				Time		Interaction Effects			
RhinAsthma Questionnaire Total Score	Treat	0.45 (1.00, ∞)	1.000	n.s	Exercise	0.47	T0	0.56	Exercise × T0	0.55	Control × T0	0.57
	Time	4.71 (1.80, ∞)	0.034	*	Control	0.52	T1	0.45	Exercise × T1	0.45	Control × T1	0.45
	Treat × Time	2.47 (1.80, ∞)	0.505	n.s			T2	0.48	Exercise × T2	0.41	Control × T2	0.56
RhinAsthma Questionnaire Limitation in Daily Life	Treat	0.12 (1.00, ∞)	1.000	n.s	Exercise	0.48	T0	0.55	Exercise × T0	0.55	Control × T0	0.56
	Time	3.36 (1.80, ∞)	0.079		Control	0.51	T1	0.46	Exercise × T1	0.47	Control × T1	0.45
	Treat × Time	1.20 (1.80, ∞)	0.960	n.s			T2	0.48	Exercise × T2	0.44	Control × T2	0.53
RhinAsthma Questionnaire Respiratory Problems	Treat	0.38 (1.00, ∞)	1.000	n.s	Exercise	0.53	T0	0.55	Exercise × T0	0.60	Control × T0	0.51
	Time	2.80 (1.82, ∞)	0.070		Control	0.48	T1	0.46	Exercise × T1	0.49	Control × T1	0.42
	Treat × Time	0.73 (1.82, ∞)	0.587	n.s			T2	0.50	Exercise × T2	0.49	Control × T2	0.50
RhinAsthma Questionnaire Rhinoconjunctivitis Score	Treat	1.81 (1.00, ∞)	0.717	n.s	Exercise	0.45	T0	0.52	Exercise × T0	0.54	Control × T0	0.50
	Time	1.95 (1.83, ∞)	0.251	n.s	Control	0.54	T1	0.45	Exercise × T1	0.37	Control × T1	0.52
	Treat × Time	4.11 (1.83, ∞)	0.058				T2	0.52	Exercise × T2	0.42	Control × T2	0.61
RhinAsthma Questionnaire Treatment and Medication Problems	Treat	0.05 (1.00, ∞)	1.000	n.s	Exercise	0.49	T0	0.55	Exercise × T0	0.56	Control × T0	0.54
	Time	4.01 (1.89, ∞)	0.044	n.s	Control	0.51	T1	0.46	Exercise × T1	0.48	Control × T1	0.44
	Treat × Time	3.80 (1.89, ∞)	0.098	n.s			T2	0.49	Exercise × T2	0.43	Control × T2	0.55
RhinAsthma Questionnaire Impairment in Sensory Perceptions	Treat	0.08 (1.00, ∞)	1.000	n.s.	Exercise	0.49	T0	0.55	Exercise × T0	0.52	Control × T0	0.58
	Time	2.98 (1.93, ∞)	0.105	n.s.	Control	0.51	T1	0.47	Exercise × T1	0.50	Control × T1	0.43
	Treat × Time	2.47 (1.93, ∞)	0.347	n.s.			T2	0.48	Exercise × T2	0.44	Control × T2	0.52
Visual Analogue Scale General Health	Treat	0.95 (1.00, ∞)	0.661	n.s.	Exercise	0.46	T0	0.44	Exercise × T0	0.32	Control × T0	0.56
	Time	2.51 (1.90, ∞)	0.169	n.s.	Control	0.53	T1	0.54	Exercise × T1	0.55	Control × T1	0.53
	Treat × Time	5.60 (1.90, ∞)	0.009	*			T2	0.51	Exercise × T2	0.51	Control × T2	0.50
Visual Analogue Scale Allergic Symptoms	Treat	0.42 (1.00, ∞)	1.000	n.s	Exercise	0.53	T0	0.46	Exercise × T0	0.38	Control × T0	0.46
	Time	7.50 (1.98, ∞)	0.002	**	Control	0.48	T1	0.59	Exercise × T1	0.65	Control × T1	0.59
	Treat × Time	10.32 (1.98, ∞)	<0.001	***			T2	0.46	Exercise × T2	0.56	Control × T2	0.46
	Treat × T1	10.55 (1.00, ∞)	0.005	**								
	Treat × T2	21.25 (1.00, ∞)	<0.001	***								

F1-LD-F1 model with time and treatment (treat, exercise or control) and the interaction of treatment and time (treat × time); T0, T1 and T2 time points T0 = day 0, T1 = day 10, T2 = day 60; *** <0.001; ** <0.01; * <0.005; <0.1; n.s. not significant.

**Table 4:** Results from the F1-LD-F1 model for the six-minute walk test.

Parameter	F1-LD-F1 Model				Relative Treatment Effects							
		F	*p*-Value				Time		Interaction Effects			
Six-Minute Walk Distance (m)	Treat	0.34 (1.00, ∞)	1.000	n.s.	Exercise	0.47	T0	0.44	Exercise × T0	0.43	Control × T0	0.43
	Time	5.61 (1.84, ∞)	0.009	**	Control	0.52	T1	0.54	Exercise × T1	0.58	Control × T1	0.58
	Treat × Time	1.18 (1.84, ∞)	1.000	n.s.			T2	0.52	Exercise × T2	0.55	Control × T2	0.55
Peak Respiratory Frequency (1/min)	Treat	0.17 (1.00, ∞)	1.000	n.s.	Exercise	0.52	T0	0.45	Exercise × T0	0.55	Control × T0	0.34
	Time	2.45 (1.86, ∞)	0.091		Control	0.49	T1	0.54	Exercise × T1	0.51	Control × T1	0.58
	Treat × Time	5.85 (1.86, ∞)	0.015	*			T2	0.51	Exercise × T2	0.49	Control × T2	0.54
Peak Minute Ventilation (L/min)	Treat	0.04 (1.00, ∞)	1.000	n.s.	Exercise	0.50	T0	0.45	Exercise × T0	0.47	Control × T0	0.43
	Time	6.07 (1.68, ∞)	0.012	*	Control	0.50	T1	0.54	Exercise × T1	0.53	Control × T1	0.55
	Treat × Time	0.67 (1.68, ∞)	1.000	n.s.			T2	0.51	Exercise × T2	0.51	Control × T2	0.51
BORG Scale Dyspnea Post-Test	Treat	0.15 (1.00, ∞)	1.000	n.s.	Exercise	0.52	T0	0.55	Exercise × T0	0.59	Control × T0	0.50
	Time	4.71 (1.83, ∞)	0.056		Control	0.49	T1	0.52	Exercise × T1	0.51	Control × T1	0.53
	Treat × Time	1.13 (1.83, ∞)	0.839	n.s.			T2	0.44	Exercise × T2	0.45	Control × T2	0.43
BORG Scale Fatigue Post-Test	Treat	0.78 (1.00, ∞)	0.577	n.s.	Exercise	0.54	T0	0.52	Exercise × T0	0.61	Control × T0	0.44
	Time	1.42 (1.76, ∞)	1.000	n.s.	Control	0.47	T1	0.52	Exercise × T1	0.54	Control × T1	0.51
	Treat × Time	2.64 (1.76, ∞)	0.234	n.s.			T2	0.44	Exercise × T2	0.46	Control × T2	0.47

F1-LD-F1 model with time and treatment (treat, exercise or control) and the interaction of treatment and time (treat × time); T0, T1 and T2 time points T0 = day 0, T1 = day 10, T2 = day 60; ** <0.01; * <0.005; <0.1; n.s. not significant.

## Supplementary Materials

The following are available online at https://www.mdpi.com/1660-4601/16/11/2040/s1, Table S1: Descriptive statistics of the exercise group. Table S2: Descriptive statistics of the control group.

## List of Abbreviations

ACT	asthma control test
FeNO	fractional exhaled NO
FEV1	forced expiratory volume in 1 second
FEV1/FVC	forced expiratory volume in 1 second of forced vital capacity
FVC	forced expiratory volume
IgE	immunoglobulin E
MEF25%–75%	mid-expiratory flow at 25%–75% of the FVC
NAL	nasal lavage
nparLD	nonparametric longitudinal data analysis
PEF	peak expiratory flow
RAST	radioallergosorbent test
RBC	red blood cell count
RTE	relative treatment effect
6MWT	six-minute walk test
