# Supplementary material for: Winter Exercise Reduces Allergic Airway Inflammation: A Randomized Controlled Study
Source: Int J Environ Res Public Health. 2019 Jun 8;16(11):2040. doi: 10.3390/ijerph16112040 (PMC6603979; doi:10.3390/ijerph16112040)
Supplement: Supplementary file 1 [file ijerph-16-02040-s001.pdf]

International Journal of Environmental Research and Public Health

MDPI

# Supplemental Materials

**Table A.** Descriptive statistics of the exercise group.

| | Day 0 | | Day 10 | | Day 60 | |
|---|---|---|---|---|---|---|
| | Exercise Group (*n* = 18) | | Exercise Group (*n* = 18) | | Exercise Group (*n* = 18) | |
| | mean ± SD | median ± IQR | mean ± SD | median ± IQR | mean ± SD | median ± IQR |
| Pulse (bpm) | 70.99 ± 12.57 | 70.38 ± 23.00 | 69.61 ± 6.98 | 69.00 ± 10.25 | 66.89 ± 9.39 | 67.50 ± 9.00 |
| BP-Systole (mmHg) | 125.28 ± 14.46 | 123.5 ± 14.50 | 126.44 ± 13.60 | 124.0 ± 22.25 | 125.72 ± 12.08 | 129.5 ± 18.25 |
| BP-Diastole (mmHg) | 76.44 ± 9.97 | 78.0 ± 15.00 | 77.83 ± 8.41 | 76.0 ± 14.50 | 79.33 ± 7.75 | 79.5 ± 8.50 |
| Oral FeNO (ppb) | 40.78 ± 23.03 | 38.0 ± 39.00 | 22.89 ± 12.42 | 21.0 ± 19.00 | 30.56 ± 24.16 | 23.0 ± 44.00 |
| Nasal FeNO (ppb) | 465.33 ± 178.07 | 451.0 ± 146.0 | 363.56 ± 195.63 | 354.0 ± 346.0 | 390.67 ± 248.82 | 367.0 ± 319.0 |
| Nasal Eosinophil Count (%) | 6.72 ± 13.43 | 1.95 ± 5.40 | 2.63 ± 3.33 | 1.31 ± 2.18 | 7.59 ± 15.02 | 1.65 ± 6.53 |
| Mucociliary Clearance Time (min) | 11.13 ± 5.44 | 15.00 ± 7.56 | 8.59 ± 5.54 | 7.21 ± 10.67 | 11.51 ± 5.39 | 15.00 ± 7.88 |
| **Spirometry** | | | | | | |
| FVC (%) | 108.17 ± 17.66 | 108.0 ± 12.75 | 108.33 ± 15.88 | 108.0 ± 10.75 | 111.50 ± 13.46 | 110.0 ± 10.25 |
| FEV1 (%) | 96.28 ± 20.98 | 98.5 ± 21.25 | 98.44 ± 18.92 | 97.5 ± 24.00 | 98.28 ± 16.58 | 97.0 ± 28.75 |
| FEV1/FVC (%) | 93.39 ± 11.81 | 95.0 ± 17.00 | 94.83 ± 10.79 | 98.0 ± 17.00 | 92.39 ± 10.75 | 94.0 ± 18.25 |
| PEF (%) | 99.50 ± 26.37 | 102.5 ± 35.00 | 99.33 ± 22.09 | 100.0 ± 25.00 | 103.17 ± 20.69 | 99.0 ± 26.75 |
| MEF 25%–75% | 73.33 ± 31.74 | 71.5 ± 33.50 | 78.61 ± 30.67 | 75.5 ± 35.75 | 73.28 ± 29.91 | 71.0 ± 32.75 |
| **Differential Blood Count** | | | | | | |
| WBC ($10^3$/µl) | 7.41 ± 1.81 | 7.20 ± 2.25 | 6.42 ± 1.30 | 6.16 ± 1.48 | 7.01 ± 1.12 | 6.92 ± 1.22 |
| RBC (106/µl) | 4.82 ± 0.37 | 4.79 ± 0.41 | 4.79 ± 0.39 | 4.79 ± 0.34 | 4.79 ± 0.36 | 4.68 ± 0.44 |
| Blood Neutrophil Count (%) | 59.81 ± 8.52 | 59.96 ± 12.95 | 57.64 ± 8.81 | 58.75 ± 13.35 | 57.37 ± 9.04 | 59.10 ± 12.98 |
| Blood Eosinophil Count (%) | 3.85 ± 2.95 | 3.35 ± 1.92 | 4.28 ± 4.85 | 2.90 ± 3.35 | 4.18 ± 3.90 | 2.95 ± 2.50 |
| **Six-Minute Walk Test** | | | | | | |
| 6MWT-Distance (%) | 104.35 ± 8.17 | 105.99 ± 10.97 | 106.13 ± 6.59 | 105.75 ± 11.25 | 105.69 ± 7.72 | 106.80 ± 11.78 |
| Peak Respiratory Frequency (1/min) | 36.66 ± 10.00 | 36.35 ± 11.40 | 33.16 ± 8.58 | 35.10 ± 6.67 | 30.71 ± 12.73 | 35.90 ± 10.50 |
| Peak Minute Ventilation (L/min) | 52.67 ± 14.27 | 50.55 ± 17.52 | 55.46 ± 12.52 | 54.30 ± 19.97 | 53.46 ± 11.11 | 53.55 ± 19.05 |
| BORG Scale Dyspnea Post-Test | 2.11 ± 1.22 | 2.0 ± 2.00 | 1.72 ± 1.10 | 2.0 ± 2.38 | 1.50 ± 1.22 | 1.5 ± 1.50 |
| BORG Scale Fatigue Post-Test | 2.22 ± 1.10 | 3.0 ± 2.00 | 1.97 ± 1.05 | 2.0 ± 2.00 | 1.78 ± 1.34 | 2.0 ± 1.00 |
| **RhinAsthma Questionnaire** | | | | | | |
| Total Score | 19.09 ± 12.20 | 15.87 ± 20.18 | 14.80 ± 10.97 | 11.27 ± 13.06 | 14.20 ± 12.70 | 7.75 ± 10.66 |
| Limitation in Daily Life | 15.80 ± 12.48 | 11.11 ± 23.89 | 12.72 ± 11.68 | 10.00 ± 18.89 | 11.98 ± 12.10 | 8.89 ± 22.78 |
| Respiratory Problems | 20.37 ± 14.24 | 16.67 ± 14.39 | 16.16 ± 14.55 | 10.61 ± 18.18 | 15.66 ± 13.60 | 12.12 ± 14.39 |
| Rhinoconjunctivitis Score | 21.69 ± 15.77 | 21.43 ± 26.19 | 11.90 ± 11.11 | 9.52 ± 13.10 | 15.61 ± 16.15 | 9.52 ± 16.67 |
| Treatment and Medication Problems | 14.44 ± 14.86 | 10.00 ± 18.33 | 11.48 ± 16.93 | 6.67 ± 13.33 | 9.26 ± 16.19 | 0.00 ± 13.33 |
| Impairment in Sensory Perceptions | 23.15 ± 17.52 | 20.83 ± 22.92 | 21.76 ± 16.94 | 25.00 ± 22.92 | 18.52 ± 17.28 | 16.67 ± 16.67 |
| **Questionnaires** | | | | | | |
| Asthma Control Test | 21.39 ± 3.57 | 22.5 ± 4.50 | 2.61 ± 2.20 | 23.0 ± 3.75 | 22.61 ± 4.09 | 24.0 ± 2.75 |
| Visual Analogue Scale–General Health | 71.66 ± 14.10 | 85.50 ± 19.00 | 81.78 ± 14.99 | 85.50 ± 6.25 | 81.06 ± 13.80 | 71.22 ± 13.75 |
| Visual Analogue Scale–Allergic Symptoms | 71.31 ± 22.17 | 74.5 ± 12.19 | 85.17 ± 20.07 | 94.5 ± 11.00 | 83.28 ± 13.24 | 86.5 ± 11.50 |

**Table B.** Descriptive statistics of the control group.

| | Day 0 | | Day 10 | | Day 60 | |
|---|---|---|---|---|---|---|
| | Control Group (*n* = 22) | | Control Group (*n* = 22) | | Control Group (*n* = 22) | |
| | mean ± SD | median ± IQR | mean ± SD | median ± IQR | mean ± SD | median ± IQR |
| Pulse (bpm) | 68.73 ± 8.81 | 67.00 ± 10.75 | 69.18 ± 7.92 | 69.50 ± 9.00 | 68.73 ± 8.58 | 69.50 ± 8.75 |
| BP-Systole (mmHg) | 118.45 ± 12.35 | 117.0 ± 20.00 | 116.32 ± 9.74 | 116.0 ± 14.00 | 115.45 ± 8.85 | 116.0 ± 12.50 |
| BP-Diastole (mmHg) | 72.95 ± 10.19 | 70.0 ± 17.75 | 70.73 ± 7.72 | 69.5 ± 9.50 | 70.77 ± 8.53 | 69.0 ± 8.75 |
| Oral FeNO (ppb) | 38.91 ± 22.30 | 36.0 ± 22.50 | 38.45 ± 26.55 | 32.5 ± 26.25 | 52.05. ± 49.94 | 39.0 ± 21.75 |
| Nasal FeNO (ppb) | 430.64 ± 187.93 | 421.0 ± 207.0 | 437.55 ± 202.33 | 390 ± 131.0 | 482.59 ± 167.78 | 475.0 ± 283.25 |
| Nasal Eosinophil Count (%) | 5.70 ± 14.43 | 1.16 ± 1.56 | 3.71 ± 4.77 | 1.53 ± 3.94 | 8.21 ± 10.84 | 3.21 ± 7.22 |
| Mucociliary Clearance Time (min) | 12.43 ± 5.67 | 13.62 ± 6.81 | 11.20 ± 5.10 | 11.75 ± 7.19 | 11.66 ± 4.59 | 15.00 ± 7.07 |
| **Spirometry** | | | | | | |
| FVC (%) | 110.32 ± 16.08 | 109.0 ± 24.50 | 110.27 ± 13.32 | 113.0 ± 20.50 | 109.14 ± 13.86 | 113.5 ± 24.25 |
| FEV1 (%) | 103.73 ± 13.21 | 99.5 ± 18.50 | 104.27 ± 11.42 | 104.5 ± 19.50 | 102.91 ± 11.62 | 103.0 ± 18.75 |
| FEV1/FVC (%) | 97.73 ± 8.17 | 99.5 ± 9.75 | 98.55 ± 8.25 | 98.5 ± 10.25 | 98.55 ± 8.57 | 98.0 ± 12.25 |
| PEF (%) | 98.95 ± 15.12 | 97.0 ± 24.75 | 105.86 ± 14.94 | 104.5 ± 25.50 | 103.64 ± 15.56 | 97.0 ± 25.25 |
| MEF 25%–75% | 87.18 ± 21.81 | 88.5 ± 35.00 | 87.68 ± 23.24 | 87.5 ± 35.00 | 86.27 ± 22.71 | 81.5 ± 36.25 |
| **Differential Blood Count** | | | | | | |
| WBC ($10^3$/µl) | 5.48 ± 1.51 | 5.38 ± 2.15 | 6.10 ± 1.41 | 6.24 ± 1.70 | 5.63 ± 1.46 | 5.89 ± 1.66 |
| RBC (106/µl) | 4.97 ± 0.85 | 4.75 ± 0.49 | 4.75 ± 0.38 | 4.62 ± 0.45 | 4.73 ± 0.36 | 4.73 ± 0.42 |
| Blood Neutrophil Count (%) | 55.55 ± 8.03 | 56.00 ± 10.38 | 56.71 ± 7.58 | 56.90 ± 9.50 | 56.53 ± 7.28 | 56.70 ± 11.02 |
| Blood Eosinophil Count (%) | 3.37 ± 1.80 | 3.15 ± 2.40 | 3.38 ± 2.05 | 3.10 ± 2.90 | 3.85 ± 2.21 | 3.30 ± 2.67 |
| **Six Minute Walk Test** | | | | | | |
| 6MWT-Distance (%) | 103.36 ± 10.49 | 102.59 ± 15.75 | 108.79 ± 9.51 | 106.64 ± 14.41 | 107.60 ± 8.74 | 107.53 ± 12.96 |
| Peak Respiratory Frequency (1/min) | 30.78 ± 4.08 | 31.10 ± 4.50 | 36.55 ± 8.11 | 34.10 ± 10.52 | 35.31 ± 7.09 | 35.70 ± 7.45 |
| Peak Minute Ventilation (L/min) | 50.64 ± 16.06 | 47.35 ± 14.85 | 56.30 ± 16.02 | 55.80 ± 17.73 | 54.97 ± 17.82 | 55.05 ± 26.07 |
| BORG Scale Dyspnea Post-Test | 1.73 ± 1.07 | 2.0 ± 1.00 | 1.95 ± 1.67 | 2.0 ± 2.38 | 1.55 ± 1.66 | 1.0 ± 1.50 |
| BORG Scale Fatigue Post-Test | 1.61 ± 1.22 | 2.0 ± 1.38 | 2.07 ± 1.91 | 2.0 ± 2.38 | 1.93 ± 1.89 | 2.0 ± 1.38 |
| **RhinAsthma Questionnaire** | | | | | | |
| Total Score | 20.28 ± 14.04 | 19.25 ± 11.34 | 15.45 ± 13.55 | 11.89 ± 15.10 | 22.23 ± 18.46 | 13.94 ± 26.98 |
| Limitation in Daily Life | 18.99 ± 18.74 | 11.11 ± 27.78 | 13.84 ± 16.83 | 7.78 ± 15.00 | 17.98 ± 18.21 | 8.89 ± 22.22 |
| Respiratory Problems | 17.91 ± 16.86 | 12.12 ± 16.67 | 13.50 ± 15.56 | 6.06 ± 18.18 | 17.36 ± 16.39 | 13.64 ± 22.73 |
| Rhinoconjunctivitis Score | 20.78 ± 18.50 | 14.29 ± 27.38 | 21.43 ± 18.34 | 16.67 ± 27.38 | 30.74 ± 26.32 | 26.19 ± 32.14 |
| Treatment and Medication Problems | 16.06 ± 18.01 | 6.67 ± 33.33 | 10.30 ± 16.93 | 0.00 ± 20.00 | 18.18 ± 22.18 | 10.00 ± 26.67 |
| Impairment in Sensory Perceptions | 27.65 ± 19.65 | 25.00 ± 22.92 | 18.18 ± 17.75 | 16.67 ± 25.00 | 26.89 ± 27.21 | 16.67 ± 31.25 |
| **Questionnaires** | | | | | | |
| Asthma Control Test | 22.86 ± 3.20 | 24.0 ± 2.75 | 23.68 ± 2.64 | 25.0 ± 1.75 | 23.32 ± 3.09 | 24.0 ± 2.00 |
| Visual Analogue Scale–General Health | 80.77 ± 17.82 | 87.50 ± 15.25 | 79.27 ± 19.50 | 85.00 ± 15.75 | 76.59 ± 22.72 | 79.50 ± 26.00 |
| Visual Analogue Scale–Allergic Symptoms | 82.45 ± 13.47 | 85.0 ± 16.50 | 83.59 ± 11.90 | 82.5 ± 19.75 | 67.50 ± 25.95 | 74.0 ± 24.25 |
